# Supplementary material for: Ribosome surface properties may impose limits on the nature of the cytoplasmic proteome
Source: eLife. 2017 Nov 20;6:e30084. doi: 10.7554/eLife.30084 (PMC5726854; doi:10.7554/eLife.30084)
Supplement: Figure 6—figure supplement 1—source data 1. [file elife-30084-fig6-figsupp1-data1.docx]

**Figure 6-figure supplement 1 source data: Free fraction of GFP variants in *E. coli*, *L. lactis* and *Hfx. volcanii*.**

| Organism | GFP variant | Mean D (µm^2^/s) | Free fraction |
| --- | --- | --- | --- |
| *E. coli* | -30 | 11 | 1 |
|  | -7 | 10 | 1 |
|  | 0 | 10 | 1 |
|  | +7 | 2.6 | 0.26 |
|  | +11a | 0.76 | 0.072 |
|  | +11b | 2.7 | 0.27 |
|  | +15 | 1.5 | 0.15 |
|  | +25 | 0.14 | 0.010 |
| *L. lactis* | -7 | 6.2 | 1 |
|  | +15 | 2.3 | 0.37 |
|  | +25 | 0.61 | 0.093 |
| *Hfx. volcanii* | -30 | 10 | 1 |
|  | -7 | 5.5 | 0.55 |
|  | +15 | 2.9 | 0.29 |
|  | +25 | 1.9 | 0.19 |
