# Supplementary material for: Ribosome surface properties may impose limits on the nature of the cytoplasmic proteome
Source: eLife. 2017 Nov 20;6:e30084. doi: 10.7554/eLife.30084 (PMC5726854; doi:10.7554/eLife.30084)
Supplement: Supplementary file 1. — (A) Amino acid sequences of the GFP variants. (B) Diffusion coefficients in E. coli, L. lactis and Hfx. volcanii. (C) P-values for pairwise comparisons of diffusion coefficients for GFP variants in E. coli (Eco), L. lactis (Lla), and Hfx. volcanii (Hvo). For E. coli we also compared diffusion coefficients under normal (0.28 Osm) and shock conditions (1.2 Osm). (D) Fitting parameters for the relation between diffusion coefficient, GFP net charge, and ionic strength. [file elife-30084-supp1.docx]

**Supplementary file 1**

**Supplementary file 1A: Amino acid sequences of the GFP variants:**

**-30 GFP**

MGHHHHHHGGASKGEELFDGVVPILVELDGDVNGHEFSVRGEGEGDATEGELTLKFICTTGELPVPWPTLVTTLTYGVQCFSDYPDHMDQHDFFKSAMPEGYVQERTISFKDDGTYKTRAEVKFEGDTLVNRIELKGIDFKEDGNILGHKLEYNFNSHDVYITADKQENGIKAEFEIRHNVEDGSVQLADHYQQNTPIGDGPVLLPDDHYLSTESALSKDPNEDRDHMVLLEFVTAAGIDHGMDELYK

**-7 GFP**

MGHHHHHHGGASKGEELFTGVVPILVELDGDVNGHKFSVRGEGEGDATNGKLTLKFICTTGKLPVPWPTLVTTLTYGVQCFSRYPDHMKQHDFFKSAMPEGYVQERTISFKDDGTYKTRAEVKFEGDTLVNRIELKGIDFKEDGNILGHKLEYNFNSHNVYITADKQKNGIKANFKIRHNVEDGSVQLADHYQQNTPIGDGPVLLPDNHYLSTQSALSKDPNEKRDHMVLLEFVTAAGITHGMDELYK
**0 GFP**

MGHHHHHHGGASKGEELFTGVVPILVELDGDVNGHKFSVRGEGEGDATNGKLTLKFICTTGKLPVPWPTLVTTLTYGVQCFSRYPKHMKRHDFFKSAMPEGYVQERTISFKDDGTYKTRAEVKFEGRTLVNRIELKGIDFKEDGNILGHKLEYNFNSHNVYITADKQKNGIKANFKIRHNVEDGSVQLADHYQQNTPIGRGPVLLPDNHYLSTQSALSKDPNEKRDHMVLLEFVTAAGITHGMDELYK

**+7 GFP**

MGHHHHHHGGASKGEELFTGVVPILVELDGDVNGHKFSVRGEGEGDATNGKLTLKFICTTGKLPVPWPTLVTTLTYGVQCFSRYPDHMKQHDFFKSAMPEGYVQERTISFKDDGTYKTRAEVKFEGDTLVNRIELKGIDFKEDGNILGHKLEYNFNSHNVYITADKRKNGIKAKFKIRHNVKDGSVQLADHYQQNTPIGRGPVLLPRNHYLSTRSKLSKDPKEKRDHMVLLEFVTAAGIKHGRDERYK
**+11a GFP**

MGHHHHHHGGRSKGKRLFRGKVPILVKLKGDVNGHKFSVRGKGKGDATRGKLTLKFICTTGKLPVPWPTLVTTLTYGVQCFSRYPKHMKQHDFFKSAMPEGYVQERTISFKDDGTYKTRAEVKFEGDTLVNRIELKGIDFKEDGNILGHKLEYNFNSHNVYITADKQKNGIKANFKIRHNVEDGSVQLADHYQQNTPIGDGPVLLPDNHYLSTQSALSKDPNEKRDHMVLLEFVTAAGITHGMDELYK

**+11b GFP**

MGHHHHHHGGASKGEELFTGVVPILVELDGDVNGHKFSVRGEGEGDATNGKLTLKFICTTGKLPVPWPTLVTTLTYGVQCFSRYPDHMKRHDFFKSAMPKGYVQERTISFKKDGKYKTRAEVKFKGRTLVNRIKLKGRDFKEKGNILGHKLRYNFNSHKVYITADKQKNGIKANFKIRHNVEDGSVQLADHYQQNTPIGDGPVLLPDNHYLSTQSALSKDPNEKRDHMVLLEFVTAAGITHGMDELYK

**+15 GFP**

MGHHHHHHGGASKGERLFTGVVPILVELDGDVNGHKFSVRGEGEGDATRGKLTLKFICTTGKLPVPWPTLVTTLTYGVQCFSRYPKHMKRHDFFKSAMPEGYVQERTISFKKDGTYKTRAEVKFEGRTLVNRIELKGRDFKEKGNILGHKLEYNFNSHNVYITADKRKNGIKANFKIRHNVKDGSVQLADHYQQNTPIGRGPVLLPRNHYLSTRSALSKDPKEKRDHMVLLEFVTAAGITHGMDELYK

**+25 GFP**

MGHHHHHHGGASKGERLFTGVVPILVELDGDVNGHKFSVRGKGKGDATRGKLTLKFICTTGKLPVPWPTLVTTLTYGVQCFSRYPKHMKRHDFFKSAMPKGYVQERTISFKKDGTYKTRAEVKFEGRTLVNRIKLKGRDFKEKGNILGHKLRYNFNSHNVYITADKRKNGIKANFKIRHNVKDGSVQLADHYQQNTPIGRGPVLLPRNHYLSTRSALSKDPKEKRDHMVLLEFVTAAGITHGMDELYK

**Supplementary file 1B: Diffusion coefficients in *E. coli*, *L. lactis* and *Hfx. volcanii*.**

| Organism | Condition | | GFP variant | N_cells_ | Mean D (µm^2^/s) | SD ^b^ (µm^2^/s) | Median D (µm^2^/s) | Q1 ^c^ (µm^2^/s) | Q3 ^c^  (µm^2^/s) |
| --- | --- | --- | --- | --- | --- | --- | --- | --- | --- |
| *E. coli* | | Normal ^a^  (0.28 Osm) | -30 | 28 | 11 | 5.2 | 10 | 6.3 | 15 |
|  |  |  | -7 | 36 | 10 | 3.4 | 10 | 8.3 | 12 |
|  |  |  | 0 | 31 | 10 | 5.3 | 8.6 | 6.4 | 12 |
|  |  |  | +7 | 39 | 2.6 | 1.1 | 2.7 | 1.9 | 3.1 |
|  |  |  | +11a | 39 | 0.76 | 0.35 | 0.67 | 0.51 | 0.90 |
|  |  |  | +11b | 35 | 2.7 | 1.4 | 2.5 | 1.9 | 3.4 |
|  |  |  | +15 | 41 | 1.5 | 0.81 | 1.2 | 0.92 | 1.6 |
|  |  |  | +25 | 70 | 0.14 | 0.061 | 0.13 | 0.088 | 0.15 |
|  |  | 0.55 Osm | -30 | 30 | 8.0 | 3.9 | 7.1 | 5.0 | 11 |
|  |  |  | -7 | 35 | 8.6 | 3.5 | 7.8 | 5.8 | 11 |
|  |  |  | +15 | 42 | 1.3 | 0.60 | 1.2 | 0.91 | 1.5 |
|  |  |  | +25 | 39 | 0.15 | 0.094 | 0.11 | 0.088 | 0.17 |
|  |  | 1.2 Osm | -30 | 23 | 0.21 | 0.15 | 0.18 | 0.10 | 0.29 |
|  |  |  | -7 | 33 | 0.50 | 0.43 | 0.36 | 0.19 | 0.75 |
|  |  |  | +15 | 31 | 0.13 | 0.12 | 0.073 | 0.050 | 0.15 |
|  |  |  | +25 | 27 | 0.049 | 0.067 | 0.020 | 0.013 | 0.051 |
| *L. lactis* | | Normal  (0.5 Osm) | -7 | 38 | 6.2 | 2.5 | 6.3 | 3.8 | 7.8 |
|  |  |  | +15 | 48 | 2.3 | 1.1 | 2.3 | 1.4 | 3.2 |
|  |  |  | +25 | 35 | 0.61 | 0.30 | 0.55 | 0.40 | 0.74 |
| *Hfx. volcanii* | | Normal  (>2.5 Osm) | -30 | 35 | 10 | 2.4 | 10 | 8.8 | 12 |
|  |  |  | -7 | 38 | 5.5 | 1.1 | 5.4 | 4.8 | 6.2 |
|  |  |  | +15 | 29 | 2.9 | 1.6 | 2.3 | 2.0 | 3.3 |
|  |  |  | +25 | 25 | 1.9 | 0.54 | 2.0 | 1.5 | 2.2 |

1. Normal means either growth medium or of the same osmolality as the growth medium.
2. SD is the standard deviation over all cells.
3. Q1 and Q3 indicate the edges of the interquartile range over all cells.

**Supplementary file 1C: *P*-values for pairwise comparisons of diffusion coefficients for GFP variants in *E. coli* (Eco), *L. lactis* (Lla), and *Hfx. volcanii* (Hvo). For *E. coli* we also compared diffusion coefficients under normal (0.28 Osm) and shock conditions (1.2 Osm).** The *p*-values were calculated using the Mann-Whitney test, and they represent the likelihood that the two compared datasets are derived from the same underlying distribution.

| Comparison | *P*-value |
| --- | --- |
| -30 GFP Hvo ↔ Eco | 0.98 |
| -7 GFP Eco ↔ Lla | 2.28*10^-7^ |
| -7 GFP Lla ↔ Hvo | 0.28 |
| -7 GFP Hvo ↔ Eco | 3.43*10^-10^ |
| +15 GFP Eco ↔ Lla | 2.02*10^-4^ |
| +15 GFP Lla ↔ Hvo | 0.11 |
| +15 GFP Hvo ↔ Eco | 7.05*10^-7^ |
| +25 GFP Eco ↔ Lla | 5.3*10^-16^ |
| +25 GFP Lla ↔ Hvo | 7.6*10^-11^ |
| +25 GFP Hvo ↔ Eco | 1.11*10^-13^ |
| Normal ↔ Shock -30 GFP | 1.16*10^-09^ |
| Normal ↔ Shock -7 GFP | 1.54*10^-12^ |
| Normal ↔ Shock +15 GFP | 6.6*10^-13^ |
| Normal ↔ Shock +25 GFP | 0.03 |
| Eco +11a ↔ +11b GFP | 4.18*10^-10^ |
| Eco -30 ↔ -7 GFP | 0.90 |
| Eco -7 ↔ +15 GFP | 6.96*10^-14^ |
| Eco +15 ↔ +25 GFP | 1.18*10^-18^ |
| Lla -7 ↔ +15 GFP | 4.51*10^-12^ |
| Lla +15 ↔ +25GFP | 5.62*10^-13^ |
| Hvo -30 ↔ -7 GFP | 2.12*10^-11^ |
| Hvo -7 ↔ +15 GFP | 2.48*10^-8^ |
| Hvo +15 ↔ +25 GFP | 3.27*10^-3^ |

**Supplementary file 1D: Fitting parameters for the relation between diffusion coefficient, GFP net charge, and ionic strength.**

| Fitting parameter | K_d_ vs ionic strength | *D_eff_* vs net charge  *E. coli* | D_eff_ vs net charge  *Hfx. volcanii* |
| --- | --- | --- | --- |
| $\boldsymbol{\Delta}\boldsymbol{G}_{\boldsymbol{nio}}^{\boldsymbol{^{\circ}}}$ (J mol^-1^) | -20 400 | -13 000 | -17 800 |
| $\boldsymbol{\Delta}\boldsymbol{G}_{\boldsymbol{pc}}^{\boldsymbol{^{\circ}}}$(J mol^-1^) | -1 156* | -2 200 | -1 150 |
| $\boldsymbol{C}_{\boldsymbol{1}}$ | 1.53 | 1.50 | 0.79 |

$*$ Calculated as follows: $\Delta G_{pc}^{^{\circ}}$=$\Delta G_{io0}^{^{\circ}}/charge$ = -28 900 J mol^-1^ / 25 = -1 156 J mol^-1^.
