# Supplementary material for: Ribosome surface properties may impose limits on the nature of the cytoplasmic proteome
Source: eLife. 2017 Nov 20;6:e30084. doi: 10.7554/eLife.30084 (PMC5726854; doi:10.7554/eLife.30084)
Supplement: Transparent reporting form [file elife-30084-transrepform.docx]

***eLife’s* transparent reporting form**

We encourage authors to provide detailed information *within their submission* to facilitate the interpretation and replication of experiments. Authors can upload supporting documentation to indicate the use of appropriate reporting guidelines for health-related research (see [EQUATOR Network](http://www.equator-network.org/%20)), life science research (see the [BioSharing Information Resource](https://biosharing.org/)), or the [ARRIVE guidelines](http://www.plosbiology.org/article/info:doi/10.1371/journal.pbio.1000412) for reporting work involving animal research. Where applicable, authors should refer to any relevant reporting standards documents in this form.

If you have any questions, please consult our Journal Policies and/or contact us: [editorial@elifesciences.org](mailto:editorial@elifesciences.org).

**Sample-size estimation**

- You should state whether an appropriate sample size was computed when the study was being designed
- You should state the statistical method of sample size computation and any required assumptions
- If no explicit power analysis was used, you should describe how you decided what sample (replicate) size (number) to use

Please outline where this information can be found within the submission (e.g., sections or figure legends), or explain why this information doesn’t apply to your submission:

The number of cells we used for each FRAP experiment is based on previous work on measuring diffusion coefficients (20-30 cells per diffusion coefficient), and larger sample sizes were not necessary to draw the conclusions from the data.

Diffusion coefficients determined for each cell were plotted in histograms to assess the distribution of the values. Histograms of FRAP results are presented in Figures 1, 2, 2-figure supplement 1, 3, 4-figure supplement 2. The numbers of cells per GFP variant in FRAP studies are reported in Supplementary file 1B. Diffusion coefficients per cell for rifampicin treated *E. coli* cells are presented in Figure 4. For colocalization of +25 GFP with DNA we indicate the number of cells in the main text.

**Replicates**

- You should report how often each experiment was performed
- You should include a definition of biological versus technical replication
- The data obtained should be provided and sufficient information should be provided to indicate the number of independent biological and/or technical replicates
- If you encountered any outliers, you should describe how these were handled
- Criteria for exclusion/inclusion of data should be clearly stated
- High-throughput sequence data should be uploaded before submission, with a private link for reviewers provided (these are available from both GEO and ArrayExpress)

Please outline where this information can be found within the submission (e.g., sections or figure legends), or explain why this information doesn’t apply to your submission:

The number of repetitions (biological replicates) is indicated in the methods section of the particular experiment or in the main text.

For the FRAP measurements of +25 GFP in *E. coli* and *Hfx. volcanii* some cells displayed heterogeneity in the distribution of fluorescence over the cytoplasm; the criteria for inclusion and exclusion of cells from further analysis as well as example images are displayed in Figure 2-figure supplement 1. For all FRAP experiments we excluded cells that were too noisy, or did not display a bleach. This is indicated in the methods section.

**Statistical reporting**

- Statistical analysis methods should be described and justified
- Raw data should be presented in figures whenever informative to do so (typically when N per group is less than 10)
- For each experiment, you should identify the statistical tests used, exact values of N, definitions of center, methods of multiple test correction, and dispersion and precision measures (e.g., mean, median, SD, SEM, confidence intervals; and, for the major substantive results, a measure of effect size (e.g., Pearson's r, Cohen's d)
- Report exact p-values wherever possible alongside the summary statistics and 95% confidence intervals. These should be reported for all key questions and not only when the p-value is less than 0.05.

Please outline where this information can be found within the submission (e.g., sections or figure legends), or explain why this information doesn’t apply to your submission:

Diffusion coefficients per cell are reported in Figure 4; in Figures 1, 2, 2-figure supplement 1, 3, 4-figure supplement 2 we report histograms of diffusion coefficients for all cells per variant. Mean, standard deviation, median, interquartile range values are reported in Supplementary file 1B. *P*-values for pairwise comparisons of diffusion coefficients are reported in Supplementary file 1C.

(For large datasets, or papers with a very large number of statistical tests, you may upload a single table file with tests, Ns, etc., with reference to sections in the manuscript.)

**Group allocation**

- Indicate how samples were allocated into experimental groups (in the case of clinical studies, please specify allocation to treatment method); if randomization was used, please also state if restricted randomization was applied
- Indicate if masking was used during group allocation, data collection and/or data analysis

Please outline where this information can be found within the submission (e.g., sections or figure legends), or explain why this information doesn’t apply to your submission:

Data is allocated to groups corresponding to GFP variants, organisms, growth conditions and specific experiments (addition of antibiotics).

**Additional data files (“source data”)**

- We encourage you to upload relevant additional data files, such as numerical data that are represented as a graph in a figure, or as a summary table
- Where provided, these should be in the most useful format, and they can be uploaded as “Source data” files linked to a main figure or table
- Include model definition files including the full list of parameters used
- Include code used for data analysis (e.g., R, MatLab)
- Avoid stating that data files are “available upon request”

Please indicate the figures or tables for which source data files have been provided:

Values of points (medians) represented in Figures 2b, 3a, 6d are available in Supplementary file 1B.

Data analysis for calculating fraction bound, K_D_ values, and the relation between diffusion coefficient, GFP net charge and ionic strength are described in the discussion and methods sections. Results of the fraction bound analysis are available in Figure 6-figure supplement 1 source data file. Fitting parameters used for the relation between diffusion coefficient, GFP net charge and ionic strength are available in Supplementary file 1D.
